# Clinicians' Experiences of Delivering Cognitive Behaviour Therapy Ten (CBT‐T): A Qualitative Investigation

**DOI:** 10.1002/erv.3200

**Published:** 2025-04-17

**Authors:** Chloe Hewitt, Siân Coker, Aaron Burgess, Glenn Waller

**Affiliations:** ^1^ Norwich Medical School University of East Anglia Norwich England; ^2^ Department of Psychology University of Sheffield Sheffield England

**Keywords:** cognitive behaviour therapy, psychological therapies, psychotherapy, qualitative research

## Abstract

**Objective:**

Cognitive Behaviour Therapy Ten (CBT‐T) is a relatively new manualised treatment for non‐underweight patients with eating disorders. It has been found to be an effective treatment and to be rated highly by patients. However, it is also important to consider clinicians' perspectives in the implementation and development of new interventions, because clinician perspectives can impact treatment delivery, leading to issues such as therapist drift. Using a qualitative approach, this research aimed to examine clinician experiences of delivering CBT‐T.

**Method:**

The sample consisted of 13 clinicians currently delivering CBT‐T, with at least six months experience of delivering this treatment. Semi‐structured interviews were conducted via Microsoft Teams, using thematic analysis to identify themes from the interview transcripts.

**Results:**

Three themes and 10 subthemes were identified. The main themes were: positive experiences of delivering CBT‐T, changing experience over time, and challenges in delivery.

**Discussion:**

Clinicians reported an overall largely positive experience of delivering CBT‐T, with some challenges related to treatment delivery identified. Findings are discussed in relation to wider research literature, with recommendations given about how clinicians can be supported with their delivery of CBT‐T, and for future research and CBT‐T development.

**Summary**

- Clinicians predominantly report positive experiences of delivering CBT‐T.
- Certain aspects of CBT‐T, particularly imagery rescripting and emotion‐focused work, are reported as areas where clinicians feel less clarity and confidence, which may influence the overall quality of treatment delivery.
- The continued development of CBT‐T should incorporate additional resources and support for clinicians in these areas. This would enable them to deliver all aspects of the treatment with maximum confidence, thereby improving treatment quality and providing patients with the best possible opportunity for recovery from their eating disorder.

## 1 Introduction

Cognitive behaviour therapy (CBT) is a widely used, evidence‐based treatment for eating disorders, recommended by National Institute for Health and Care Excellence (NICE) (National Institute for Health and Care Excellence 2017) guidelines as a treatment for all eating disorder diagnoses. However, given the physical risks associated with anorexia nervosa (Puckett et al. 2021), individuals with this diagnosis often require prioritisation for treatment (e.g., Harrop et al. 2021; Lebow et al. 2015). This can mean that those with a non‐underweight eating disorder, such as bulimia nervosa, binge eating disorder, or other specified feeding or eating disorders, often have to wait a long time to access treatment (Waller et al. 2019).

**FIGURE 1:**
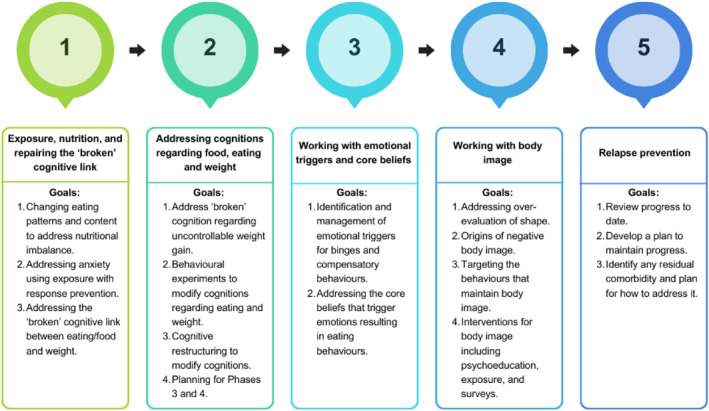
The structure of CBT‐T.

Cognitive Behaviour Therapy Ten (CBT‐T) is a manualised 10‐session therapy divided into five phases. It was developed by Waller et al. (2019) specifically for non‐underweight eating disorders to address the difficulties that these individuals can have when attempting to access treatment. Due to its brief duration, CBT‐T reduces treatment time, allowing clinicians to treat more patients in the same time period. Additionally, because CBT‐T can be delivered by clinicians without a professional qualification (under supervision), it is more cost‐effective, enabling a higher number of patients to be seen more quickly and at lower cost per head. An outline of the structure of CBT‐T and each phase of treatment is shown in Figure 1. Initial case series of CBT‐T have shown it to be an effective treatment (Pellizzer et al. 2019a, 2019b; Waller et al. 2018).

Hoskins et al. (2019) identified that in addition to investigating the effectiveness of new therapies, it is also necessary to consider how patients experience therapy, in order to aid with enhancing treatment acceptability and minimising treatment attrition. This has led researchers to investigate patients' experiences of receiving treatment, with Hoskins et al. (2019) investigating how patients experience CBT‐T. They found the acceptability and effectiveness of CBT‐T to be highly rated by patients. Key themes identified through a thematic analysis of patient responses were the therapeutic relationship, the nature of therapy, that CBT‐T was challenging but beneficial, ending therapy, and the overall experience of CBT‐T. Patients reported their experiences of this treatment as being mostly positive.

Hoskins et al. (2019) noted that their work would be enhanced by comparing it to themes derived from an analysis of clinicians' experiences of CBT‐T. Previous research has identified that it is also important to consider clinicians’ views of delivering treatment when implementing new interventions (Greenhalgh et al. 2004). It has been suggested that combining both clinician and patient perspectives provides a more in‐depth understanding of treatment, informing both the future development of interventions and clinician training (Waterman‐Collins et al. 2014).

Consideration of clinicians' experiences also enables an understanding of how clinicians can be better supported in some of the challenges they may encounter when delivering interventions (Carayon et al. 2019). It is also important to understand how clinicians experience delivering the core components of treatment, as this is likely to influence treatment fidelity and delivery. For example, Shafran et al. (2009) found that clinicians' views of treatment can affect adherence to treatment delivery, which can lead to therapist drift. This is problematic, particularly if a clinician is delivering a manualised treatment such as CBT‐T.

Understanding clinicians' views and experiences of treatment is important for a number of reasons as outlined above, but is yet to be considered for CBT‐T. As it is known that clinicians tend to offer more CBT sessions than recommended for patients with eating disorders (Cowdrey and Waller 2015), a concern was that clinicians might view CBT‐T as less acceptable than longer forms of eating disorder treatment, and therefore regard it as inappropriate or inadequate for such patients. Understanding clinician experiences could inform ongoing development of CBT‐T, and ascertain whether there is any additional support that clinicians may benefit from when delivering this therapy to ensure full adherence to the treatment manual. It would also allow direct comparison of patient and clinician experiences of CBT‐T, providing a more in‐depth understanding of this treatment. This would be helpful as well as pertinent to the current development of a shorter treatment for underweight eating disorders.

The aim of this research is to investigate clinicians' experiences of delivering CBT‐T and to answer the following research questions.

1. What are clinicians' experiences of delivering CBT‐T?
2. Are there any aspects of CBT‐T where clinicians may benefit from additional support when delivering this intervention?

## 2 Method

### 2.1 Participants

To be eligible to take part in this research, participants had to be currently delivering CBT‐T and have been delivering this therapy for at least 6 months. The criteria for currently delivering CBT‐T was to ensure an accurate reflection of current experiences rather than retrospective accounts, which have previously been found to affect the accuracy of reporting of personal experiences (Coughlin 1990; Solga 2001). The criteria of at least 6 months experience delivering CBT‐T was to ensure participants had a sufficient period of time to experience delivering this therapy.

Data saturation was used to determine the sample size, recognised as the 'gold standard' for qualitative research (Guest et al. 2006). While literature on the required sample size varies in its recommendations, studies suggest that a range of 12–15 participants is generally sufficient (Clarke and Braun 2013; Fugard and Potts 2015; Guest et al. 2006).

Seventeen individuals expressed an interest in participating. Three of these were identified to be fraudulent individuals responding to the social media advertisement. One individual who made an expression of interest and was identified as eligible for participation did not return their consent form. This left a final sample of 13 National Health Service (NHS) eating disorder clinicians. Recruitment ceased when data saturation was reached (i.e. when no new theme or subthemes emerged from the last three interviews).

Of the 13 participants, 84.62% were female (*n* = 11), with a mean age of 28.31 years at the time of interview (SD = 5.38). Participants had been delivering CBT‐T for an average of 16.54 months (SD = 10.01) and had treated a mean number of 20.38 patients (SD = 18.46) with CBT‐T. One participant had a core profession, with the remaining participants working under supervision as unqualified practitioners. Table 1 details the demographic information of all participants.

**TABLE 1:** Participant demographics.

Participant number	Age (years)	Gender identity	Ethnicity	Profession	Duration delivering CBT‐T (months)	Number of people treated with CBT‐T
P1	24	Female	White British	Clinical Associate Psychologist	30	20
P2	26	Female	White British	Assistant Psychologist	25	12
P3	24	Female	White British	Assistant Psychologist	7	9
P4	24	Female	White British	Community Practitioner	12	17
P5	27	Non‐binary	White Greek	Assistant Psychologist	7	6
P6	29	Male	White British	Assistant Psychologist	22	50
P7	27	Female	White Canadian	Assistant Psychologist	12	4
P8	26	Female	White British	Assistant Psychologist	24	60
P9	28	Female	White British	Psychology Practitioner	18	42
P10	38	Female	White British	CBT Therapist	7	4
P11	30	Female	White British	Trainee Clinical Associate Psychologist	9	7
P12	24	Female	British Pakistani	Psychology Practitioner	6	14
P13	41	Female	White British	Eating Disorder Specialist	36	20

### 2.2 Ethical Considerations

Ethical approval for this research was granted by the University of East Anglia's Faculty of Medicine and Health Sciences Research Ethics Committee in December 2022 (Ref: ETH2223‐0245) and the Health Research Authority (Project ID: 321,360) in April 2023. Participants were informed that their participation was voluntary, and that their interview transcript and personally identifying information would be anonymised. Participants were asked to create a unique patient identifier in the case of wanting to withdraw their data, and were advised that they could contact the researcher to withdraw their data should they wish to do so, up until the point that data analysis had commenced. No participants requested withdrawal of their data.

### 2.3 Procedure

Participants were recruited by emailing gatekeepers for four NHS eating disorder services known to deliver CBT‐T a recruitment poster and information about the research, asking them to disseminate this information to clinicians within the service. The recruitment poster was also shared on Twitter and Facebook. Individuals expressing an interest in taking part in this research were sent an information sheet and consent form. There were 17 people who expressed an interest in participating, with 13 people returning a signed consent form. Upon receipt of a completed consent form, participants were contacted and a convenient time for interview was arranged. Interviews were semi‐structured, using a topic guide. Using the topic guide, an initial interview schedule was developed by the authors, then shared with five qualified clinicians with experience of delivering CBT‐T for feedback. Amendments were made to the schedule on the basis of this, with the finalised interview guide comprising of 10 questions relating to various aspects of the experience of delivering CBT‐T. After piloting the interview schedule with the first three participants, it was adjusted to allow time at the end for them to share any additional thoughts about their experience delivering CBT‐T that they felt had not been addressed during the interview but wished to mention. All interviews took place via Microsoft Teams between July 2023 and October 2023, each lasting approximately one hour. Upon completion of the interview, participants were emailed a debrief form and were given a £10 gift card as a token of appreciation for their participation.

### 2.4 Data Analysis

Interview transcripts were analysed using Braun and Clarke's (2006) six‐step process of thematic analysis. This is a form of analysis used for ‘developing, analysing and interpreting patterns across a qualitative dataset, which involves systematic processes of data coding to develop themes’ (Braun and Clarke 2022, 4). Reflexive thematic analysis (Braun and Clarke 2019) is one form of thematic analysis, emphasising the importance of critical reflection on being a researcher. This approach was felt to be important in this research given the researchers' own experiences of delivering CBT‐T and the potential impact of that experience upon the research.

Thematic analysis aligned with the critical realist positioning of this research and compliments the analysis in the research exploring patient experiences of receiving CBT‐T. Alternative qualitative methods were considered, but thematic analysis was deemed most suitable for this research. For example, interpretative phenomenological analysis focuses on individual experiences, while this study explored broader perspectives. Grounded theory was unsuitable as the study did not aim to develop hypotheses or theories. Given that CBT‐T is a relatively new treatment with limited research on clinicians' experiences, thematic analysis ensures that their perspectives are captured without over‐interpretation, which could be a risk with discourse analysis.

Interviews were live transcribed using Otter, an artificial intelligence transcription software offering live transcription at the time of interview, and which is compliant with General Data Protection Regulation (GDPR) rules. Transcripts were checked by author CH for accuracy, with any personally identifying information removed. Interview recordings were listened to at least twice in line with the guidance by Braun and Clarke (2022) to aid with familiarisation of transcripts. Transcripts were printed, with content relevant to the research questions (i.e. content referring to participants' experience of delivering CBT‐T or aspects of CBT‐T delivery that may require additional development) coded by hand. These codes were then collated into potential themes and subthemes. Themes were reviewed both independently and through the use of supervision, with themes defined and named once these had been finalised. A reflective diary was kept throughout the research process to aid reflexivity and to record the rationale for key decision points throughout the research.

## 3 Results

From analysis of interview transcripts, three main themes and 10 subthemes were identified. The themes, subthemes and examples of quotations are shown in Table 2.

**TABLE 2:** Themes, subthemes, and example quotations.

Main themes	Sub‐themes	Example quotations
1. Positive experiences of CBT‐T delivery	1a. Enjoyable to deliver	‘A really nice therapy to deliver and work with patients on’ (P13) ‘A safe therapy [to deliver]’ (P1) ‘[I have] liked delivering CBT‐T the most out of all therapies’ (P2) ‘I'm massively passionate about it. It's a really lovely therapy’ (P13)
	1b. Strong therapeutic alliance	‘I feel like it's [the therapeutic alliance] been really good. I Think it tends to be strong’ (P1) ‘It's something that maybe I thought would be more difficult with CBT‐T just because of various elements that are quite different to the way I was working before’ (P2) ‘Definitely still managed to build that relationship’ (P7) ‘If they can do the changes then the therapeutic alliance is going to be stronger’ (P1)
	1c. The treatment protocol	‘[It] is containing…having that guidance and knowing what to do’ (P9) ‘You know you're delivering the right thing that you're meant to deliver each week’ (P11) ‘It was really helpful in those early days’ (P6)
	1d. Good experience for patients	‘Largely positive’ (P8) ‘By the end [patients] feel really positive about things’ (P1) ‘People always seem to say they feel more confident leaving the sessions, and I think that says a lot about how powerful CBT‐T is’ (P6)
2. Changing experience over time	2a. Reduced anxiety and increased confidence	‘I became more and more confident with delivering it’ (P5) ‘As time goes on I get more confident’ (P4) ‘Got more confident with what I'm doing’ (P11) ‘My anxiety has come down over time and my need to almost over‐prepare for each session has been reduced’ (P2)
	2b. Belief in model	‘Seen that it can work’ (P1) ‘Really know and appreciate what people can get from that’ (P6) ‘I've seen the positive outcome that it has’ (P3) ‘Putting it into practice helps firm up my understanding’ (P4)
3. Challenges in delivery	3a. Better fit for some patients than others	‘Works really well when you get people who have certain presentations’ (P9) ‘When I see people who have binge eating disorder or bulimia, for me they've done really well’ (P12) ‘Sometimes with people who would be described as complex, I find I really struggle to address everything in 10 sessions’ (P5) ‘If they're very early within the eating disorder, say it's only been 2 years, it can be difficult to get past that stage [Phase 1]’ (P12)
	3b. Areas requiring additional support	‘[Imagery rescripting is something I would] like more training on’ (P4) ‘Imagery rescripting [in the manual] isn't detailed enough to feel comfortable delivering it as a clinician’ (P12) ‘[Emotion work] is where the manual is a bit vague…I stray and bring other stuff in’ (P9) ‘[The manual] says that you can use some DBT skills, but for somebody who has never really used CBT before it doesn't really explain what that might look like in the context of CBT‐T’ (P2) ‘[I'm unsure] how you address some of these emotions and different ways to cope with them’ (P7)
	3c. Terminating treatment	“It feels really hard to be sat in front of another human, not as a patient and as a therapist, but as a human being saying, ‘you've not done enough to receive any more of our help’” (P3) ‘It feels difficult not to be able to offer them more time’ (P6) ‘Sometimes the person thinks they're on track to keep going and wants to keep going and we don't feel the same, and that's the difficult part’ (P9) ‘The hardest times have been when people are very keen, and they want help but have struggled to make the change’ (P8)
	3d. Treatment infidelity	‘Definitely had to make adaptations’ (P7). ‘If you're going by the protocol you're not supposed to slow it down or make adaptations, but with a real person we, in my service at least, often find that we have to’ (P9). ‘Don't stop at the eating disorder’ (P5) ‘It's hard not to veer out of it [the protocol] sometimes when other stuff comes up” (P11). “We have a thing known as session zero, which is like a mini assessment to get to know the person that you're going to work with’ (P4).

The first theme highlights the predominantly positive experience participants reported in delivering CBT‐T. They expressed generally favourable attitudes toward providing CBT. Participants noted their ability to establish strong therapeutic alliances with patients, with some expressing their surprise at being able to do so. Some identified that they are able to build stronger therapeutic alliances with patients who are finding CBT‐T beneficial. Participants found having a treatment protocol to be beneficial as clinicians, and believed that CBT‐T offers a positive experience for patients.

The second theme reflects the way that participants' experience of delivering CBT‐T has changed over time. Participants reflected on their early experiences of delivering CBT‐T and compared them to their feelings after gaining more experience over time. They noted that as they became more familiar with the intervention, their anxiety about delivering it decreased, and their confidence grew. Additionally, they identified that their belief in CBT‐T as a treatment has been enhanced through delivering it, particularly from seeing its effectiveness.

The third theme notes the range of challenges noted when delivering CBT‐T. A consistent challenge participants identified from their experiences is feeling that the CBT‐T is better suited to some patients and presentations than others, citing binge eating disorder and bulimia nervosa as specific diagnoses where they have seen CBT‐T to be most effective. Comorbidity and complexity were also raised as factors affecting the suitability of CBT‐T. Participants also talked about the parts of CBT‐T they found most difficult to deliver; both the phase three imagery rescripting and emotion work were raised by almost all participants interviewed. They described that additional support or training with these would help them to feel more comfortable and confident with delivering these interventions. It was noted by participants that they found it difficult to implement boundaries in terms of terminating treatment. Furthermore, many examples were given by participants about times where they have not adhered to the treatment protocol. These ranged from additional sessions to including material from outside CBT‐T and addressing comorbid problems within treatment. Participants were consciously aware that they were deviating from the CBT‐T protocol.

## 4 Discussion

The aim of this study was to investigate clinicians' experiences of delivering CBT‐T using semi‐structured interviews and thematic analysis. The study was designed to compliment the earlier research investigating patient experiences of receiving CBT‐T (Hoskins et al. 2019). In the current research, the themes that were identified from the interviews were positive experiences of CBT‐T delivery, the changing experience of delivering CBT‐T over time, and the challenges that arise when delivering CBT‐T. Clinicians were largely positive about their experience of delivering CBT‐T, describing it as enjoyable and finding it useful to have a protocol to follow. They also talked about having been able to build strong therapeutic alliances with their patients within the short timeframe of CBT‐T, and feeling that the treatment was a positive experience for patients. Clinicians identified that their experience of delivering CBT‐T changed over time, specifically with regards to their confidence using CBT‐T and their belief in the treatment model. Challenges identified by participants were that they felt that CBT‐T was a better fit for some patients than others, and that they found it difficult to terminate treatment when this is required. Participants also gave examples of when they have moved away from the treatment manual, with additional support needs identified for delivering the emotion work and imagery rescripting.

Participants becoming more confident with delivering CBT‐T over time is unsurprising and is unlikely to be limited to the delivery of any specific therapy (e.g., Bischoff et al. 2007; Nurse et al. 2025). The same can be said for participants' belief in the CBT‐T model being enhanced from seeing the effectiveness. However, what is more unexpected is the value that participants placed on the CBT‐T treatment manual. Previous research largely indicates that clinicians often have unfavourable opinions towards manualised treatments (Addis and Krasnow 2000; Muskat et al. 2010; Waller et al. 2013). In the current study, whilst there were indications that some participants found using a manualised treatment limiting, on the whole participants were positive about using a treatment manual. The results of a systematic review by Forbat et al. (2015) found that clinician views on manualised treatment can be affected by age, years of experience, gender, race, and educational background, with research by Addis and Krasnow (2000) also finding a relationship between less experienced clinicians and more positive attitudes towards manuals. Considering this finding, it is important to note that the majority of participants in this research were of a similar age and did not have a professional qualification. This also inhibited exploration of experiences of delivering CBT‐T in comparison to other therapies, as most participants did not have the experience of delivering other interventions. Future research may helpfully address this limitation.

The previous research by Hoskins et al. (2019) showed patients' ratings of the therapeutic alliance in CBT‐T to be high, with the current study demonstrating that clinicians' perspectives on this are complimentary. Within clinical practice there are two opposing views regarding the therapeutic alliance. The first is that building a therapeutic alliance is essential for change (Baier et al. 2020; Beck 1979), whereas others suggest that the therapeutic alliance develops as a result of early change and patients seeing that treatment works (Tang et al. 2007; Waller et al. 2012); CBT‐T aligns with the latter. One of the specific stipulations within the CBT‐T manual is that there should not be a focus on developing a therapeutic alliance at the cost of progressing the tasks of therapy. Accordingly, time should not be specifically allocated to building a therapeutic alliance, and instead the focus should be on supporting patients to make changes from the start of treatment, with change emphasised from session one. Despite this, the findings of both this study and aforementioned research indicate that both patients and clinicians describe the therapeutic alliance in CBT‐T to be strong. This aligns with the findings of a previous meta‐analysis by Graves et al. (2017) which found early symptom improvement to be related to the subsequent quality of the therapeutic alliance. This has implications theoretically with regards to the theory underlying the development and importance of the therapeutic alliance in regards to change. There are also key clinical implications in terms of how this is applied in clinical practice, as this finding indicates that it is still possible to build a good therapeutic alliance without allocating specific time to this.

It was clear from the identification of the subtheme ‘treatment infidelity’ that clinicians were not always delivering CBT‐T in accordance with the protocol guidelines. Given that therapist drift is reported to be a common phenomenon across psychological treatments (Waller 2009; Waller and Turner 2016), this is perhaps unsurprising. Nevertheless, it is problematic as it results in patients receiving treatment that moves away from the evidence‐base, potentially impacting treatment outcomes. Previous research has identified that there can be a range of reasons for therapist drift including therapist anxiety (Hernandez Hernandez and Waller 2021; Moritz et al. 2019), clinical experience (Beidas et al. 2014; Sijercic et al. 2020), therapist knowledge (Becker‐Haimes et al. 2019; Sars and van Minnen 2015), therapist age (Mulkens et al. 2018; Wisniewski et al. 2018), and theoretical orientation (R. de Jong et al. 2020; Garcia et al. 2020). Although this research identified that clinicians were not consistently adhering to the treatment protocol, a limitation of the current research is that reasons for this were not explored. If it is possible to identify and understand why clinicians are not adhering to the protocol, this will enable any additional guidance or support to be developed, as appropriate, to improve treatment fidelity.

Emotion work and imagery rescripting were identified as the main areas that participants reported that they need additional support with implementing. Reasons for this appear to be related to participants feeling that this is not sufficiently covered within the CBT‐T training and treatment manual. Accordingly, a recommendation of the current research is that CBT‐T training should more explicitly cover these areas, and for the manual to provide clearer guidance on these areas. Participants in the current research indicated that if they had more understanding of these areas then they would be more likely to explore these with patients, which could enhance patients' experiences of treatment as a result. Given that role play is indicated to enhance learning and understanding (Flaherty 2023; Issac Gibbs 2019), training could include the opportunity to role play these interventions to aid with improving clinician understanding of and confidence with using these. The results of this research also indicate that future editions of the CBT‐T manual should include clearer guidance on how to address the areas of emotion work and imagery rescripting within the 10‐session delivery. Research has indicated that clinicians' views on manualised treatment improve with such protocol amendments made in accordance with their feedback (Stith et al. 2002; Taylor et al. 2011), so making these changes might also contribute to further enhancement of clinicians' overall experiences of delivering CBT‐T. It would be of benefit to re‐investigate clinicians' experiences of CBT‐T after these changes have been made as a way of evaluating the impact with respect to both clinician experiences and the effects on their delivery of CBT‐T.

A strength of the current research is that this is the first study to investigate clinicians' experiences of delivering CBT‐T, complimenting the earlier research on patients' experiences of receiving this form of therapy. However, it is worthwhile noting some of the limitations of this research. The recruitment method could have introduced a source of bias in that those who were well disposed towards CBT‐T were more likely to volunteer for this research, possibly influencing the positive experiences reported. This approach to recruitment might have led to an underrepresentation of individuals with less enthusiasm or who have had more negative experiences with CBT‐T, as they may have been less motivated to participate. As a result, the positive experiences reported might reflect the views of those more aligned with CBT‐T, rather than offering a broader, more diverse perspective on clinicians' experiences. Likewise, as previously discussed, the age and relative inexperience of some of the participants, both with delivering therapeutic interventions and CBT‐T specifically, may have also influenced the findings. Furthermore, although this research focused specifically on the delivery of CBT‐T, it may be possible that some of the findings are more generally applicable to therapeutic interventions as a whole. In addition, two of the authors are experienced in the delivery and supervision of CBT‐T, and one of these was lead author on the manual. These experiences might have introduced biases towards positive experiences of delivering CBT‐T, and identifying these as being core themes. A reflective diary and regular supervision was used to mitigate this risk. Finally, it should be acknowledged that the current research did not consider the potential link between patient outcomes and clinician experiences, whereby clinicians might report more positive experiences of delivering CBT‐T if they believe that their patients are benefitting from treatment. Future studies could examine this possible relationship more closely to address this limitation.

In conclusion, the current research found clinicians report largely positive experiences of delivering CBT‐T, with a number of components identified to contribute to this. This mirrors previous findings on patient experiences of this treatment, indicating that CBT‐T is seen as an acceptable treatment by both clinicians and patients. There are areas of CBT‐T that clinicians report feeling less clear about and confident in delivering, namely imagery rescripting and emotion work, which may affect the quality of treatment delivery (Bartle‐Haring et al. 2022; Seewald and Rief 2023). The current research suggests that the ongoing development of CBT‐T should incorporate additional information about and support for clinicians in these areas to allow them to maximally and confidently deliver all elements of this intervention, enhancing treatment delivery and allowing patients the best possible chance to recover from their eating disorder.

## Ethics Statement

Ethical approval for this study was obtained from the Health Research Authority (Project ID: 321,360) and the University of East Anglia's Faculty of Medicine and Health Sciences Research Ethics Committee in December 2022 (Ref: ETH2223‐0245).

## Conflicts of Interest

Professor Glenn Waller receives royalties on the CBT‐T manual, however we know of no other conflicts of interest associated with this publication.

## Data Availability

The data that support the findings of this study are available from the corresponding author upon reasonable request.
